# In Vivo Approaches to Understand Arrhythmogenic Cardiomyopathy: Perspectives on Animal Models

**DOI:** 10.3390/cells13151264

**Published:** 2024-07-27

**Authors:** Giovanni Risato, Raquel Brañas Casas, Marco Cason, Maria Bueno Marinas, Serena Pinci, Monica De Gaspari, Silvia Visentin, Stefania Rizzo, Gaetano Thiene, Cristina Basso, Kalliopi Pilichou, Natascia Tiso, Rudy Celeghin

**Affiliations:** 1 Department of Cardio-Thoraco-Vascular Sciences and Public Health, University of Padua, I-35128 Padua, Italy; giovanni.risato@unipd.it (G.R.); marco.cason@unipd.it (M.C.); maria.buenomarinas@unipd.it (M.B.M.); serena.pinci@studenti.unipd.it (S.P.); monica.degaspari@unipd.it (M.D.G.); s.rizzo@unipd.it (S.R.); gaetano.thiene@unipd.it (G.T.); cristina.basso@unipd.it (C.B.); kalliopi.pilichou@unipd.it (K.P.); rudy.celeghin@unipd.it (R.C.); 2 Department of Biology, University of Padua, I-35131 Padua, Italy; raquel.branascasas@phd.unipd.it; 3 Department of Women’s and Children’s Health, University of Padua, I-35128 Padua, Italy; silvia.visentin.1@unipd.it

**Keywords:** arrhythmogenic cardiomyopathy, animal models, mouse, zebrafish, heart

## Abstract

Arrhythmogenic cardiomyopathy (AC) is a hereditary cardiac disorder characterized by the gradual replacement of cardiomyocytes with fibrous and adipose tissue, leading to ventricular wall thinning, chamber dilation, arrhythmias, and sudden cardiac death. Despite advances in treatment, disease management remains challenging. Animal models, particularly mice and zebrafish, have become invaluable tools for understanding AC’s pathophysiology and testing potential therapies. Mice models, although useful for scientific research, cannot fully replicate the complexity of the human AC. However, they have provided valuable insights into gene involvement, signalling pathways, and disease progression. Zebrafish offer a promising alternative to mammalian models, despite the phylogenetic distance, due to their economic and genetic advantages. By combining animal models with in vitro studies, researchers can comprehensively understand AC, paving the way for more effective treatments and interventions for patients and improving their quality of life and prognosis.

## 1. Introduction

**Figure 1:**
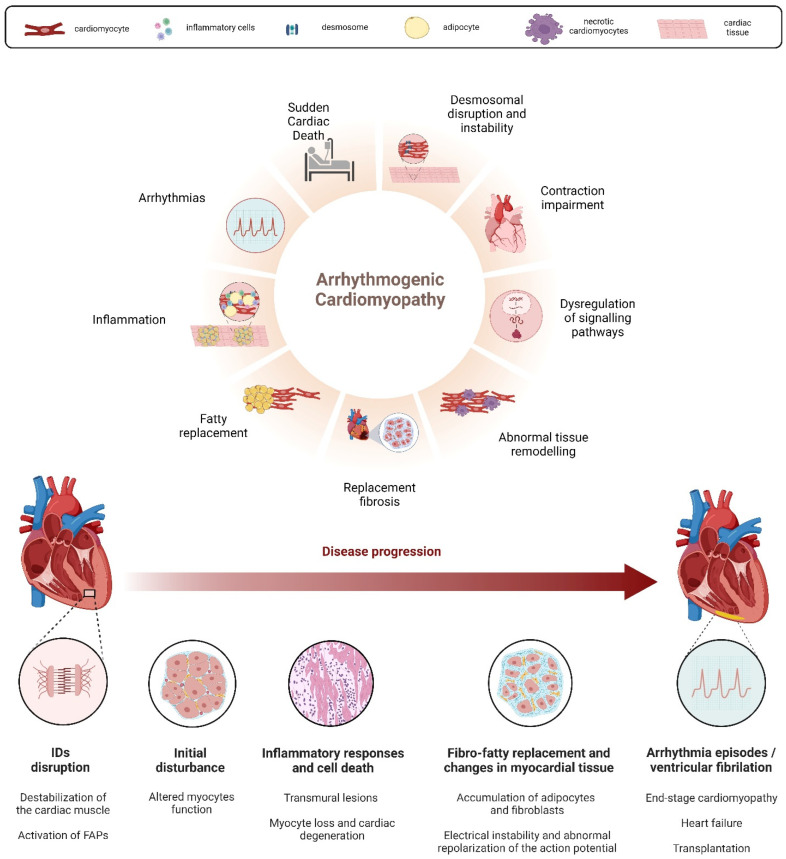
Hallmarks of AC disease pathogenesis and progression detected in human patients. The central hallmark of AC is the fibro-fatty substitution of the myocardial tissue, being linked to the instability/disruption of intercalated discs (IDs) and desmosomes, the activation of FAPs (fibroblast activation proteins), the dysregulation of signalling pathways such as Wnt/β-catenin, and inflammation. Inflammatory infiltrates are associated with necrotic and/or apoptotic myocyte death, leading to electrical instability, arrhythmias, and SCD. Image created with BioRender.

**Figure 2:**
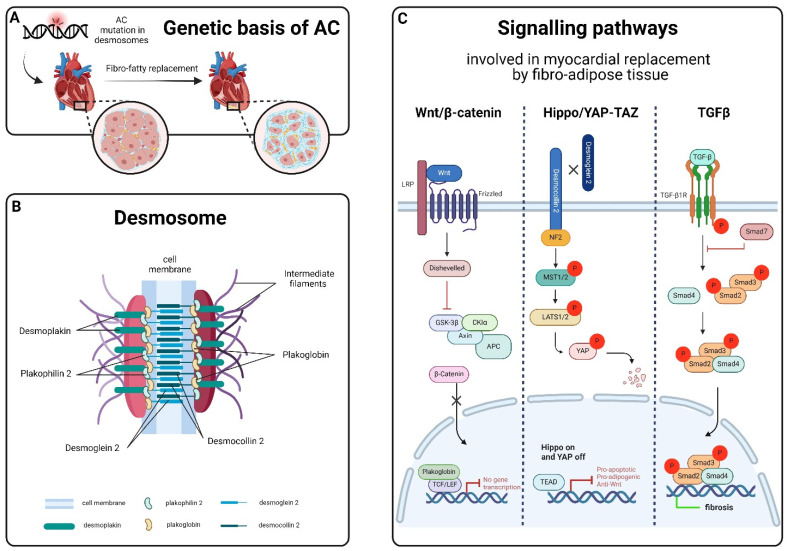
Genetic and Signalling pathway dysregulation in AC. (**A**): Desmosomes play a crucial role in cell adhesion, mechanical anchorage, and communication between cardiomyocytes. (**B**): The desmosome structure consists of several proteins: Desmoplakin (DSP), Plakoglobin (JUP), and Plakophilin-2 (PKP2) form the intracellular portion, separated into an outer and inner dense plaque, while the intercellular portion is characterized by the interaction between cadherin proteins, Desmoglein-2 (DSG2), and Desmocolin-2 (DSC2). (**C**): The dysregulation of signalling pathways is fundamental in the progression of the disease. Wnt/β-catenin dysregulation occurs through desmosome destabilization and subsequent JUP protein release, leading to adipogenesis and fibrogenesis. The activation of the Hippo/YAP-TAZ pathway leads to the accumulation p-YAP in the cytosol, its degradation and the subsequent altered gene expression associated with adipogenesis and apoptosis. p-YAP also binds and sequesters β-catenin, reducing its transcriptional activities. The upregulated TGF-β signalling pathway contributes to cardiac repair, remodelling, and fibrosis. Image created with Biorender.

Arrhythmogenic cardiomyopathy (AC) is an inherited cardiac disorder characterized by fibrous and adipose tissue that gradually replaces cardiomyocytes, beginning at the epicardium and progressing to the endocardium [1,2]. This transmural damage results in myocardial atrophy, with possible wall thinning and aneurysms usually in the right ventricle, and accounts for the clinical presentation with syncope, palpitations, ventricular arrhythmias, and impaired ventricular systolic function. In many cases, it culminates in sudden cardiac death (SCD) in young individuals, including athletes [1,2,3,4]. Notably, physical exercise and competitive sports can trigger life-threatening ventricular arrhythmias, accelerating disease progression and the risk of SCD in AC patients [5]. The prevalence of AC in the general population is approximately 1:2000–5000 [3,4,6,7], although this frequency might be underestimated due to diagnostic challenges [8]. Males are affected up to three times more frequently than females, a disparity linked to sex-related differences in exercise volume and intensity or the physiological effects of sex hormones [9,10,11,12]. The fibro-fatty substitution of the myocardial tissue, the main hallmark of AC, appears to be linked to the instability and disruption, driven by genetic factors, of desmosomes’ structure [1,2,13] (Figure 1). Desmosomes, symmetrical multi-protein structures within intercalated discs (IDs), play a critical role in cell adhesion, mechanical anchorage and communication between cardiomyocytes, facilitating force transmission and maintaining electrical continuity [14,15,16,17]. These complexes also contribute to apoptosis, electrochemical coupling, tissue differentiation, and cell-to-cell communication [18]. The desmosome structure consists of cytoplasmic proteins (plakin and armadillo families) and transmembrane sticky glycoproteins (cadherin superfamily). Desmoplakin (DSP), Plakoglobin (JUP), and Plakophilin-2 (PKP2) form the intracellular portion, separated into an outer and inner dense plaque, while the intercellular portion is characterized by the interaction between cadherin proteins, Desmoglein-2 (DSG2) and Desmocolin-2 (DSC2) [19,20] (Figure 2). Ventricular arrhythmias in AC result from pathological changes in myocardial tissue, causing inhomogeneity, altered conduction, and the abnormal repolarization and depolarization of the action potential [2,13,21]. AC patients often experience periodic inflammatory cells infiltrates in the myocardium, which resemble acute responses like those seen in myocarditis or myocardial infarction and could accelerate the progression of the disease [2,8,22,23,24]. Necrotic and/or apoptotic myocyte death is implicated in the cascade of events leading to fibro-fatty replacement, possibly connected to AC pathogenesis [25,26,27]. In end-stage cardiomyopathy, myocyte loss occurs due to both apoptosis and necrosis, contributing to the progression of cardiac dysfunction and degeneration [25,27,28,29]. Treatments are mainly focused on SCD by using antiarrhythmic drugs, beta-blockers, implantable cardiac defibrillators (ICDs), and catheter ablation; heart transplantation is applied either for refractory life-threatening arrhythmias or more rarely for end-stage heart failure [30]. However, these cures are palliative; thus, ongoing research aims to develop preventive therapies based on newly identified biomarkers [31], addressing the genetic nature of AC.

### 1.1. Genetics of AC

AC is primarily considered a heterogeneous inherited cardiomyopathy transmitted as an autosomal dominant trait with incomplete penetrance and variable expressivity. Compound/digenic heterozygotes and homozygous recessive forms have also been described as severe forms of the disease, associated with almost complete penetrance and cutaneous abnormalities [32,33,34,35]. About 40% of affected individuals carry mutations in genes encoding desmosomal proteins. However, for more than half of AC patients, a definitive genetic cause has not been identified, suggesting an oligogenic inheritance or complex genomic rearrangement. Currently, mostly desmosomal genes have been definitively proven to be associated with AC; all other genes either have strong or moderate evidence or have been recently published with limited data and identified in few families with the AC phenotype [36].

### 1.2. Signalling Pathways Related to AC

The disruption and instability of desmosomes in AC contribute to the dysregulation of signalling pathways involved in the myocardial replacement by fibro-adipose tissue, such as Wnt/β-catenin, Hippo/YAP-TAZ, and TGFβ [37,38,39]. The Wnt/β-catenin and Hippo/YAP-TAZ pathways, in particular, play a crucial role in cardiac development, controlling proliferation, differentiation, tissue remodelling, and apoptosis [40,41,42,43,44,45,46]. Recent studies have linked the dysregulation of Wnt/β-catenin signalling to AC through desmosome destabilization and subsequent JUP protein release, leading to adipogenesis and fibrogenesis [37,45,47]. Dysregulated activation of Hippo/YAP-TAZ in AC, instead, results in the remodelling and instability of IDs, leading to elevated levels of phosphorylated YAP (p-YAP) and altered gene expression associated with adipogenesis and apoptosis [48,49]. Furthermore, p-YAP has been identified to suppress the Wnt/β-catenin pathway by binding and sequestering β-catenin, reducing transcriptional activities [45]. The TGF-β signalling pathway, upregulated in AC patients, contributes to cardiac repair, remodelling, and fibrosis [50,51]. Excessive or prolonged signalling alteration exacerbates unfavourable remodelling, promoting myofibroblast trans-differentiation and facilitating the transition to scar formation [42,52,53].

### 1.3. Search Strategies and Selection Criteria

Search engines in the medical literature included PubMed/Medline and Scopus, using the following keyword search strings: “AC” OR “ACM” OR “Arrhythmogenic cardiomyopathy” AND “animal models” OR “Mice” OR “Zebrafish”. We carefully reviewed reference lists of original publications and review articles for missing studies. Duplicates were eliminated. All studies were filtered independently by 3 reviewers (GR, RC, and MC), and occasional disagreements were settled by additional authors (NT, KP, and CB). Only original peer-reviewed and review articles providing accurate animal model phenotypic characterizations were considered. The ZFIN database was used to check the availability of not-yet-characterized zebrafish lines.

## 2. Animal Models for AC

**Figure 3:**
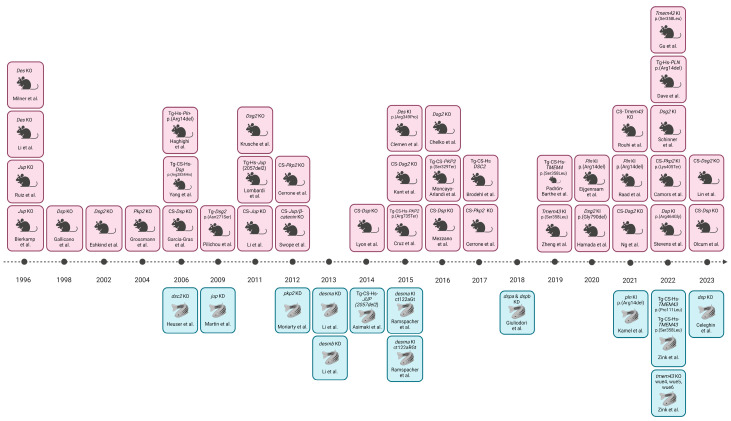
Timeline overview of in vivo animal models for AC-related genes, organized by the year of publication. Abbreviations: CS = cardiac-specific; Tg = transgenic; Hs = *Homo sapiens*; KD = knock down; KO = knock out; KI = knock in. Updated from Gerull and Brodehl, 2020 [75]. Image created with Biorender.

Animal models have significantly enhanced our comprehension of human diseases at the cellular and molecular levels, offering insights into pathophysiology, disease progression, and the impact of environmental factors, while also aiding in the development and testing of novel therapeutic approaches. Over the past decade, scientists have established a range of in vivo models incorporating targeted genetic mutations and/or the transgenic overexpression of AC-related genes [19,54]. Notably, instances of spontaneous AC manifestation have been observed in domestic animals, including a case in a primate [55]. Moving to other animals, particularly boxer dogs and related English bulldogs [56], instances of cardiomyopathy were commonly associated with the disease [57]. Mutations causing AC in boxer dogs were found triggering significant alterations in the mechanical and electrical connections between cardiomyocytes [58]. AC also affects other dog breeds, including Fila Brasileiro dogs [59], Springer Spaniel dogs [60], Labrador retrievers [61], Weimaraner dogs [62], Shetland sheepdogs, Dalmatians [63], and Siberian huskies [64]. In feline models, clinically significant cardiomyopathies closely resembling the human condition have been discovered, with marked myocardial injury, fibrous and/or fatty replacement, myocarditis, and apoptosis [65,66,67]. While these feline models exhibit all the common clinical characteristics of AC, their entirely spontaneous nature poses limitations for examining the pathophysiological process from the onset of AC. To address this limitation, laboratory animal models, such as mice or zebrafish with genetically induced diseases have been developed, overcoming the constraints of spontaneous models. The mouse (*Mus musculus*) is a widely utilized animal model for studying human diseases due to the substantial similarities between the human and murine genomes. Approximately 99% of human genes have direct orthologues in mice, and there are notable parallels in their morphology, cell biology, and physiology. Over the last 15 years, the mouse model has particularly emerged as an excellent organism for researching human cardiac diseases. The zebrafish (*Danio rerio*) has emerged as a valuable alternative to mice due to its ease of manipulation and biological relevance. Rodent studies, often marked by cost, time, and ethical constraints, can be addressed by leveraging zebrafish, which share orthologs for approximately 71% of human proteins, with 82% linked to human diseases [68,69]. The structural and electrical similarities between zebrafish and human hearts make *Danio rerio* an effective model for understanding cardiac genes associated with various cardiovascular diseases, potentially facilitating the discovery of new treatments [70,71,72,73]. Despite these advantages, zebrafish, like mice, struggle to exhibit adipogenic substitution in cardiac tissue. Notably, adult zebrafish cardiomyocytes display proliferative and regenerative capacities after injuries, resembling features seen in the early postnatal mammalian ventricle [74]. Mice and zebrafish models with mutations in AC-related genes have been developed in past years, employing various strategies such as gene knock down (KD) and knock out (KO), transgenic overexpression (Tg), or knock-in (KI) of human AC-gene variants. Among these methods, KD/KO-mediated gene inactivation is widely utilized to model loss-of-function mutations (Figure 3). 

## 3. Genes Definitively Associated with AC

### 3.1. Plakoglobin

The N- and C-terminal domains of JUP are connected by a central region that displays highly conserved armadillo repeats (12 arm repeats), which are typical of this protein family [76]. JUP demonstrates two distinct locations: adherent junctions, where it can be replaced for the closely related armadillo protein β-catenin (CTNNB1), and desmosomes, where it interacts strongly with the cytoplasmic domains of DSG2 and DSC2 cadherins in the outer dense plaque. By interacting with DSP, JUP connects the inner dense plaque to the extracellular portion of the desmosome, forming a bridge [77]. On the Greek island of Naxos in 1986, Protonotarios and colleagues linked JUP to a recessive form of AC for the first time [32]. According to a 2007 study by Asimaki et al., an in-frame insertion of a serine residue p.(Ser39_Lys40insSer) at the N-terminus of the JUP protein has been associated with the dominant form of AC in a small German family [78]. Less than 1% of AC patients carry *JUP* pathogenic variants [41,78,79] but this seems significantly higher in specific geographic regions [80].

***Mouse.*** In 1996, the central role of JUP in the stability of the desmosomal structure in both heart and skin tissues was demonstrated in vivo for the first time. Specifically, a *Jup* homozygous null-mutant mouse model showed significant embryonic lethality due to severe heart defects. The surviving embryos exhibited a reduced number of desmosomes and developed cardiac dysfunction, with a larger right ventricle, a higher frequency of spontaneous ventricular arrhythmias, and slower right ventricular conduction. No abnormalities in CX43 distribution and localization were observed, and there was no replacement fibrosis or remodelling of the junctions. Heterozygous animals appeared to be quite healthy, showing no cardiac structural defects [81,82]. However, right ventricular dilation, decreased function, and ventricular arrhythmia were detected. *Jup* mutated mice mimicked the cutaneous phenotype in humans, with skin blistering and subcorneal acantholysis. Swimming endurance training hastened the onset of ventricular failure and arrhythmias in these animals, while a reduction in the ventricular load and training prevented the development of AC [83,84]. Cardiac-specific (CS)-*Jup* ablation in mice resulted in a human AC phenotype condition with increased dilatation, fibrosis, cell death events and SCD, without evidence of myocardial fat [85]. To better understand the role of β-catenin (CTNNB1), a double CS-*Jup*/*Ctnnb1* KO mouse model was generated, demonstrating the crucial role of both proteins in preserving ID structures and mechano-electrical coupling. Indeed, this model showed clear conduction problems, fibrous tissue replacement and spontaneous ventricular arrhythmia, all leading to SCD. The ID structures were collapsed with a reduced presence of CXC43 in gap junction plaques, as in AC human hearts [86]. A Tg mouse line with a cardiac overexpression of human Naxos-associated truncated JUP protein presented premature mortality, heart dysfunction and fibro-adiposis. This truncated form had less membrane localization and decreased binding with DSP and DSG2 proteins [87]. The normalization of the levels of JUP in both these models alleviated the condition, providing a possible novel therapeutic approach for AC [88]. Epidermal growth factor receptor (EGFR) suppression was assessed under both healthy and pathological conditions as a possible therapeutic approach, surprisingly improving cardiomyocyte cohesion. This revealed a direct interaction between EGFR and DSG2 [89]. 

***Zebrafish***. The initial strategy to analyze AC-related genes in zebrafish employed KD techniques. A *jup* KD using antisense morpholino oligomers induced an immediate cardiac phenotype characterized by edema, a reduced heart size, blood reflux and a twisted tail. This pathological manifestation correlated with dysregulated Wnt/β-catenin signalling, the reduced localization of desmosomes and adherens junctions in the IDs [90]. Subsequently, Asimaki and colleagues (2014) established a Tg zebrafish line expressing the human *JUP* (2057del2) mutation responsible for Naxos syndrome, recapitulating a similar phenotype. The *jup*-mutant zebrafish model exhibited abnormal cardiac physiology, penetrant cardiomyopathy marked by atrial and ventricular wall thinning, pericardial effusion, and mortality due to arrhythmias or heart failure. A high-throughput pharmacological screen on this mutant line identified SB216763 as a suppressor of the disease phenotype, reducing mortality and preventing heart failure [38]. This study demonstrated the utility of zebrafish models in uncovering novel AC mechanisms and identifying mechanism-based drugs to alleviate AC characteristics. 

### 3.2. Desmoplakin

DSP, the most prevalent protein in desmosomes, mediates the interaction between the intermediate filaments (IFs) and the plasma membrane and is necessary for continuous adhesion [91]. DSP is a member of the plakin family and consists of three domains: a central α-helical coiled-coil domain (rod domain) involved in protein dimerization, a globular N-terminal domain important for localization and heterophilic protein–protein interactions including those with PKP2, a C-terminal domain made up of three plakin repeat domains (A-B-C), and a glycine-serine-arginine rich domain (GSR) that directly interacts with IFs [92]. Two isoforms of DSP are produced by alternative mRNA splicing, differing in the length of the central rod domain: DSP I, primarily expressed in the heart with 2871 amino acids, and DSP II, with only 2271 amino acids. Recently, DSP II was found in both ventricular and atrial tissues, challenging fundamental assumptions about AC once again [91]. Carvajal syndrome was the first autosomal recessive form of cardiomyopathy found in South America and associated with mutations in *DSP*, characterized by keratoderma, woolly hair, and AC [33,93]. The first autosomal dominant pathogenic variants found in the *DSP* gene, located on the short arm of chromosome 6 (6p24.3), were a heterozygous nonsense variant, p.(Gly331Ter), and a splicing site variant, c.939+1G>A [94]. Later, an autosomal dominant heterozygous missense variant p.(Ser299Arg) in the *DSP* gene was shown to be present in six AC-affected individuals from an Italian family living in the Veneto Region [95].

***Mouse***. The constitutive homozygous deletion of *Dsp* in mice provoked premature embryonic death even before the evaluation of a possible cardiac phenotype [96]. To address this issue, a cardiac specific (CS-*Dsp* KO) mouse line was generated by using αMHCcre induction. This model in heterozygosity showed early ultrastructural defects in desmosomes, cardiac dysfunction, cell death, inflammation, ventricular arrhythmias, an excess of adipocytes and fibrosis in the myocardium, SCD and Hippo/YAP-TAZ and Wnt/β-catenin signalling pathways’ dysregulation. Homozygous CS-*Dsp*-KO mice died at embryonic stages with only a partially formed heart and no chamber specification [37,97,98]. In a cardiac conduction system-specific *Dsp* ablated mouse model, a clear connection between this protein and the regulatory electrical activity of the heart was observed [99]. Moreover, a new autosomal recessive *Dsp* mutation called Ruffled (rul) was recently found causing an abnormal hair coat along with the classic AC condition, strictly resembling the human Carvajal-Huerta Syndrome [33,100,101]. In 2019, Malireddi et al. proposed a novel form of cell death called PANoptosis, characterized by pyroptosis, apoptosis and necroptosis, but that cannot be explained by any of them on its own [102,103]. Mutant CS *Dsp*-KO mice presented this new cell death phenomenon as a prominent phenotypic feature, associated with all pathological events described before [104]. In these mice with *Dsp* deficiency exclusively confined to cardiomyocytes, the severity of the pathological phenotype is intensified by exercise, and cardiomyocytes showed electrical problems linked to CX43 expression in both homozygous and heterozygous backgrounds [105,106]. Although not lethal, the cardiac overexpression of a mutant *DSP* cDNA with a C-terminal mutation p.(Arg2834His) led to impaired ventricular function, dilated ventricles, and elevated apoptosis with fibrosis. An altered connection between DSP and JUP was also observed [107]. Endurance exercise protocols and the related cardiovascular stress accelerate AC pathogenesis in both overexpressing and KI/KO mouse models, revealing electrical and structural abnormalities, as well as perturbed GSK3-β signalling [105,106,108,109].

***Zebrafish***. Morpholino injections targeting the *DSP* orthologs *dspa* and *dspb* in zebrafish caused desmosome structure destabilization, mirroring human patients’ phenotypes [39]. Altered signalling pathways associated with AC, such as Wnt/β-catenin, TGFβ/Smad3, and Hippo/YAP-TAZ, were identified. The drug SB216763, previously effective in a *jup*-KI zebrafish model [38], was later tested in the *dsp* double KD model, confirming its rescuing effects [39]. Recently, our group investigated the relationship between DSP and AC phenotypes in zebrafish, generating a stable *dsp*-KO zebrafish model. They followed the progression of the disease from the larval stage to adulthood and, for the first time, confirmed that AC can be faithfully mimicked in all its phases in fish as in mice [110]. Due to the duplication of the *dsp* gene, the study focused on the double heterozygous *dspa/dspb* combination, both being members expressed in the heart [39], in this way mimicking the heterozygosity commonly found in humans. At the larval stage, this model presented an early AC-related phenotype, with cardiac alterations, edema, and the accumulation of inflammatory cells in the cardiac region. The ventricle ejection fraction, contractility, and heart rhythm were altered, with a significant detection of arrhythmic events. The histological examination of mutated adult hearts revealed diminished contractile structures, an irregular ventricle shape, myocardial layer thinning, dilated vessels, and the presence of adipocytes in the myocardium. Additionally, “pale”, disordered, and delocalized desmosomes were found by Transmission Electron Microscopy (TEM) examination. An intense physical training programme accelerated the disease’s progression by exacerbating the cardiac phenotype at both early and late stages of the disease. Dysregulation of several signalling pathways, among which was Wnt/β-catenin, prompted the use of the Wnt/β-catenin agonist SB216763 to mitigate the phenotype. This treatment rescued the pathway expression as well as the cardiac abnormalities, stabilizing the heart rhythm and reducing the frequency of the arrhythmic episodes, confirming Wnt/β-catenin as a potential therapeutic target for this disease [110]. 

### 3.3. Plakophilin-2

PKP occurs in many forms (PKP1-3), with PKP2 being expressed mostly in heart tissue [111]. It is a structural protein interacting with desmosomal cadherins and DSP [112]. The insertion of 44 amino acids between arm repeats 2 and 3 characterized PKP2 isoforms, transcript 2b (881 amino acids) and 2a, exclusively expressed in the heart (837 amino acids) [113,114]. The core region of the protein contains nine arm repeats with a flexible loop in the middle, between the fifth and sixth arm repeats. *PKP2* pathogenic variants were first identified in a Dutch AC cohort by Cox and colleagues [79]. 

***Mouse***. The homozygous deletion of the *Pkp2* gene caused embryonic fatal changes in heart morphogenesis, characterized by decreased trabeculation, an unorganized cytoskeleton, ruptures in the cardiac walls and blood release into the pericardial cavity. DSP was observed to be separated from the desmosome structure, and was instead aggregating in the cytoplasm; no defects were detected in the skin tissue. Therefore, a heterozygous mouse strain was used, although only electrical but not histological signs of the disease were detected [115]. In the hearts of heterozygous CS-*Pkp2* KO mice, a reduced number of sodium channels and altered IDCs were associated with sodium dysfunction and arrhythmias [116,117]. In addition, Camors et al. observed in a KI mouse model, expressing a human truncating variant of PKP2, p.(Lys405Ter), characterized by the insertion of thymidine in exon 5, which mimics a familial case of AC in humans, a reduced actin expression and a related lack of ventricle contraction [118]. A training protocol can create a pro-arrhythmogenic state in a CS-*Pkp2*-induced conditional KO [119,120] and, specifically, the Tg overexpression of the human *PKP2* truncated mutation p.(Arg735Ter) resulted in an exercise-dependent AC phenotype with a clear right ventricle dysfunction [121]. A cardiac overexpression of human *PKP2* variant p.(Ser329Ter), instead, resulted in a phenotype like the one described in the CS-*Pkp2* mutant mouse model, with a CX43 delocalization but no fibro-fatty replacement [122].

***Zebrafish***. Morpholino-mediated KD of PKP2 in zebrafish embryos induced cardiac edema, incomplete heart looping, reduced heart rate and a twisted tail, akin to the *jup*-KD model [90,123]. The cardiac desmosome structure was altered, and co-injection of wild type (WT) *pkp2* mRNA rescued the morpholino-induced (“morphant”) phenotype [123].

### 3.4. Desmoglein-2

Four different DSGs with a highly similar structure (DSG 1-4) are expressed in different tissues and facilitate calcium-dependent cell–cell adhesion [124]. DSG2 is present in all tissues harbouring desmosomes, even though it appears to be the only isoform expressed in cardiac tissue [125,126]. DSG2 is composed of four extracellular cadherin domains: a transmembrane domain, an intracellular anchoring domain, a tiny signal domain known as RUD (repeated-unit domains), whose function is still unknown, and a calcium-binding site that stabilizes each domain. *DSG2* was first connected with AC in 2006 [127], with the discovery of 9 missense variants affecting highly conserved amino acids in 8 Italian families presenting a clear pathological phenotype [127].

***Mouse.*** *Dsg2*-KO mutant mice display cardiomyocyte loss and fibrotic and hypertrophic cardiac remodelling. Only 30% of *Dsg2*-KO homozygous mice survived the embryonic stage [128,129,130,131,132]. Moreover, the *Dsg2* KI mutant mice exhibited premature death during swimming activities and environmental stresses, displaying myocardial dysfunction, necrosis, inflammation, calcium overload and mitochondrial apoptosis [133,134,135,136]. Additionally, pharmacological inhibition of *GSK3β* (a Wnt/β-catenin modulator) improved cardiac function [133,137]. In CS-*Dsg2* KO the heart function was significantly compromised in response to rising mechanical demands, with prominent morphological abnormalities [138]. Cardiomyocyte mechanical stress sets off an early immune response and tissue remodelling in the heart at a later stage in a CS-KO of *Dsg2* [139]. Lin et al. demonstrated lipid accumulation and heart failure in their CS-*Dsg2* KO mouse model, caused by impaired mTOR-4EBP1-PPARα-dependent fatty acid β-oxidation. Adjusting PPARα activity alleviated the pathological phenotype, suggesting it as a potential target for AC [140]. Tg mice with a cardiac overexpression of p.(Asn271Ser) mutation were generated to investigate its role in the disease, directly paralleling the human AC mutation p.(Asn266Ser). Notably, a reduction in desmosomal structures with “pale” and non-compact IDs was associated with SCD events. Additional phenotypes included ventricular dilatation, aneurysms, conduction slowness, spontaneous ventricular arrhythmias, myocyte necrosis, inflammation, and fibrous tissue generation [27,127]. A reduced action potential (AP) velocity was also observed, demonstrating a direct interaction between DSG2 and the sodium channel protein Na(V)1.5 [141]. The results mirrored those observed in *Dsg2*-KO mice [129,131,138]. A *Dsg2*-KI mouse model, eliminating the tryptophan exchange (W2A) crucial for DSG2 interactions, displayed a severe cardiac profile with arrhythmia, cardiac fibrosis and decreased systolic function. Altered integrin-αVβ6 and TGFβ signalling, along with decreased fibrosis and pro-fibrotic marker expression upon the integrin-αVβ6 blockade, were observed [142]. A treatment with extracellular vesicles (EVs) in a homozygous KI mutant *Dsg2* mouse decreased cardiac inflammation and arrhythmia episodes, with a global enhancement of cardiac function [24].

### 3.5. Desmocollin-2

There are three known isoforms of DSC (DSC 1- 3), although only DSC2 is expressed throughout the body and identified in cardiac tissue [124]. The protein structure consists of four extracellular domains that are extremely conserved, the first of which is called CAR and is in charge of the heterophilic contact with the DSGs in neighbouring cells. A transmembrane domain connects them to an intracellular anchor domain at the N-terminus, which binds the intracellular portion of the desmosome in the inner dense plaque, making up the structure of DSC2. Four probands affected by AC were discovered to carry heterozygous *DSC2* frameshift variants [143]. Since then, less than fifty *DSC2* nucleotide variants, which account for 1–3% of AC cases, have been identified [41,79,143,144]. 

***Mouse.*** KI mice p.(Gly790del) did not show any sign of AC [145], whereas the cardiac overexpression of WT DSC2 in mice was shown to induce necrosis, acute inflammation and cardiac fibrotic remodelling leading to cardiomyopathy [146].

***Zebrafish***. Heuser et al. utilized morpholino oligomer injections to KD *dsc2* expression in zebrafish, resulting in cardiac edema, decreased fractional shortening and altered desmosomal structures. The model exhibited reduced desmosomal plaque area, loss of extracellular electron-dense midlines and associated myocardial contractility abnormalities. This underscores the essential role of the *dsc2* gene in normal myocardium development in both zebrafish and humans [144].

### 3.6. Transmembrane Protein-43

Transmembrane Protein-43 (TMEM43) is essential to the inner nuclear membrane. It is composed of a broad hydrophilic domain that is exposed to the endoplasmic reticulum (ER) and four transmembrane domains (TMDs). It has been demonstrated that TMEM43 interacts with emerin and lamins, two elements of the linker of nucleoskeleton and cytoskeleton (LINC) complex, and is therefore implicated in the organization of the nuclear membrane [147,148,149]. *TMEM43* was added to the pool of genes strongly correlated with AC in recent years [36]. The TMEM43 founder mutation p.(Ser358Leu) was discovered in a well-characterized AC community in Newfoundland and was later confirmed in populations in the UK, Denmark, Germany, and Spain. The *TMEM43* founder variant causes a severe sex-influenced fatal AC that results in left ventricular dilatation, fibro-fatty replacement, heart failure and SCD [150,151,152,153]. 

***Mouse.*** A *Tmem43* CS-KO mouse line showed an age-dependent phenotype characterized by an increased mortality, cardiac dilatation and dysfunction, myocardial fibrosis, adipogenesis, and apoptosis [154]. In contrast, the KI mouse model carrying the p.(Ser358Leu) presented with gender-specific cardiac dysfunction and the dysregulation of signalling pathways, such as Wnt/β-catenin and PPARG signalling, as in humans. The Wnt/β-catenin alteration was validated by the translocation of JUP into the nuclei of mutant cardiomyocytes. Although the systolic dysfunction appeared earlier in homozygous mutant mice, stress test intolerance was observed in both genetic combinations, accompanied by arrhythmias and fibro-fatty infiltration. In the cardiac tissues of these mice, NF-κB activation was present, boosting downstream signalling and the expression of pro-fibrotic TGFβ1 [155,156,157]. Moreover, Padrón-Barthe and colleagues demonstrated that administering a GSK3β inhibitor to Tg mice overexpressing *TMEM43* in either its WT or p.(Ser358Leu) mutant form enhanced heart function and activity [158]. The same model treated with Enalapril, an ACE inhibitor, showed reduced fibrosis, improved ECG and echocardiographic parameters, and increased survival [158,159].

***Zebrafish.*** In a *tmem43*-KO zebrafish model, significant ventricular enlargement was observed only in adulthood, with no overt cardiac abnormalities or contractile dysfunction during early embryogenesis stages were observed. To further explore this aspect, Tg CS zebrafish lines overexpressing human WT *TMEM43* and two genetic variants, p.(Ser358Leu) and p.(Pro111Leu), were created. Heterozygosity in this line resulted in mTOR pathway activation, ribosome biogenesis, and enlarged hearts with cardiomyocyte hypertrophy, cardiac morphological defects at juvenile stages, and ultrastructural changes within the myocardium, accompanied by dysregulated gene expression profiles in adulthood [149].

## 4. Genes Strongly or Moderately Associated with AC

### 4.1. Phospholamban

The *PLN* gene codes for the 52-amino acid protein Phospholamban, located in the sarcoplasmic reticulum (SR) membrane. Sarco/endoplasmic reticulum Ca^2+^-ATPase (SERCA), which moves calcium from the cytosol into the SR, is primarily regulated by PLN, which is a key player in cardiomyocyte calcium management. In its dephosphorylated form, PLN inhibits calcium absorption by reducing SERCA’s affinity for Ca^2+^. When PLN is phosphorylated at serine 16 by protein kinase A (PKA) or at threonine 17 by Ca^2+^/calmodulin-dependent protein kinase II (CaMKII), the PLN-mediated inhibition of SERCA is relieved, leading to an increase in SERCA activity and calcium uptake. Heart contraction and relaxation depend on the PLN-SERCA interaction, which is controlled by the β-adrenergic receptor pathway to adjust cardiac output to physiological demands [160,161]. A Greek family affected by hereditary heart failure was found to be a carrier of the c.40_42delAGA variant, a heterozygous deletion of arginine 14 p.(Arg14del) [162]. Remarkably, this pathogenic variant was identified in about 14% of Dutch patients with AC and DCM, being classified as a founder mutation in the Netherlands [163,164,165,166,167,168,169].

***Mouse.*** Mice harbouring the human mutation p.(Arg14del) exhibited increased arrhythmias, ventricular action potential prolongation, unresponsive to β-adrenergic stimulation, and electric remodelling with affected calcium homeostasis and dysregulation of Sarco(endo)plasmic reticulum Ca^2+^-ATPase (SERCA) activity [161,164,170,171,172]. Specifically, the mutant PLN protein is localized at the plasma membrane and modifies the activity of the Na/K ATPase (NKA), failing to co-localize with SERCA2 [162,164]. Interestingly, in Tg mice, the disruption of the human allele by the AAV9-CRISPR/Cas9 approach strongly improved cardiac function, providing preclinical evidence for therapeutically suppressing the AC phenotype in these AC patients [173].

***Zebrafish***. Adult zebrafish carrying the p.(Arg14del) variant manifested tissue remodelling with sub-epicardial inflammation, fibrosis and adipogenic substitution. Echocardiography revealed contractile variations correlating with action potential duration alternance at the cellular level. Calcium level alterations were detected at both embryonic and adult stages. Treatment with Istaroxime, a calcium regulator, improved cardiac relaxation, restored cellular action potential duration and mitigated calcium dysregulation [174].

### 4.2. Desmin

Desmin is connected to many cellular structures such as desmosomes, Z-bands, mitochondria, and nuclei, and is located in the cytoskeleton of cardiomyocytes. Its role is connected to preserving the structural integrity of cardiomyocytes [175]. Mutations in this protein can disrupt subcellular organelle organization, lead to the development of inclusion bodies, weaken the DES cytoskeleton, and ultimately cause myofibril disintegration. Heterozygous missense or in-frame deletion mutations account for most pathogenic *DES* variants, causing abnormal filament assembly and aggregation [176,177]. A case report by Otten et al. in 2010 described two Dutch families with characteristics resembling AC. These patients experienced muscular weakness and cardiac arrhythmias both in early childhood and later in life [178].

***Mouse.*** Both KO and KI p.(Arg349Pro) mice developed a cardiac phenotype with fibrosis, arrhythmias, protein aggregates, mitochondrial and conductions defects, partially mimicking AC [179,180,181,182]. In the homozygous *Des*-KO mouse model, coagulation and complement system activation interacted, exacerbating myocardial injury and impairing sinoatrial pacemaker function. This alteration was mitigated by using the thrombin inhibitor Lepirudin [183,184,185,186].

***Zebrafish.*** Morpholino antisense oligomers were used to KD both orthologs *desma* and *desmb* in zebrafish. These morphant lines developed cardiac edema, arrhythmias, and dysfunctions, resulting in decreased viability [187]. Furthermore, Ramspacher and colleagues characterized two KI zebrafish models (ct122aGt and ct122aRGt) in 2015, revealing that the loss of Desmin function promotes skeletal muscle defects, alters heart biomechanics and affects contraction [188].

## 5. Non-Desmosomal Genes with Disputed, Limited, or Not Curated Evidence of Disease Association

Non-desmosomal genes with disputed, limited, or not curated evidence of association with AC have been listed in Table 1.

## 6. Conclusions and Limitations

Progressive fibro-fatty substitutions of the myocardium, leading to life-threatening electrical instability and eventually ventricular dysfunction, are characteristic features of AC. Despite ongoing research using various techniques, fully replicating AC’s clinico-pathological characteristics in laboratory settings remains challenging. Developing more sophisticated AC models is crucial for comprehensively understanding its pathophysiology. Therefore, human clinical evidence should drive the outcomes and derivable searches of these models. In vitro cell models, extensively discussed and summarized in other reviews [231,232,233], while valuable and quicker to generate than animal models, have limitations related to their origins, genetic factors, maturity, cell type, and environmental interactions, lacking the complexity of whole organs. One of the main in vitro cardiac models is the HL-1 cell line, which is able to spontaneously contract while maintaining a differentiated cardiac phenotype [37,48,234,235,236,237,238,239,240,241,242]. These cells have also been used to test drug responses [89,243]. Ventricular cardiomyocytes from neonatal rats (NRVM) cell lines have similarly elucidated AC mechanisms, confirming findings in HL-1 cells [134,244,245,246,247]. Neonatal rat ventricular fibroblasts (NRVFs) are also very useful to study cytoskeleton organization, elasticity, and cell–cell adhesion properties [248]. Additionally, the extraction of cardiomyocytes from genetically modified zebrafish hearts, as performed in rat and mice, is very useful for in vitro investigations [38]. Zebrafish models have also been used to create 3D in vitro cardiac cultures [249]. Fibro-adipocyte progenitors cells (FAPs) preferentially differentiated to adipocytes if Wnt signalling was dysregulated due to a DSP loss of function mutation [250]. Although all these animal-derived cell models provide a valuable and easily accessible source of functional cells, validation in human-derived models is required. Non-contractile cardiac mesenchymal stromal cells (C-MSCs) isolated from AC patients allow for the study of their involvement in the disease’s adipogenic substitution and myofibroblast differentiation [52,251,252,253,254,255]. Buccal mucosa cells express elevated levels of all isoforms of heart desmosomal proteins, making them an important alternative to cardiomyocytes [256,257,258]. Keratinocyte cultures from skin biopsies mirror cardiac desmosomal protein expression, localization, and mutations, validating their use in AC studies [259,260,261]. HEK293 cells also present all human protein post-translational modifications, high transfection efficiencies, quick growth, and an efficient and adaptable metabolism [78,262,263,264,265,266,267,268]. Human induced pluripotent stem cells (hiPSCs) can serve as a valuable resource for obtaining human-derived cardiomyocytes (hiPSC-CMs) with patient-specific genome characteristics, unlimited availability, and suitability for high-throughput screening, allowing the study of AC protein mutations [233,269,270,271,272,273,274,275,276,277,278,279,280,281,282,283,284,285,286,287]. Although various in vitro alternative models have been explored, their main drawback is the absence of the complex microenvironment found in heart tissue, including interactions among different cell types such as fibroblasts, immune cells, vascular cells, cardiac progenitors, and cardiac myocytes. These interactions are essential for the mechanical and electrical properties of cardiac tissue, contributing to tissue morphogenesis, differentiation, and homeostasis. Moreover, addressing the progressive nature of AC is crucial when discussing its limitations in cell models. Therefore, in vivo animal models play a fundamental role in validating and delving deeper into the observations made in the various in vitro cell models briefly described above. Over the past two decades, efforts have been made to overcome these limitations by generating and characterizing numerous in vivo models. Various genetic approaches have been employed to investigate gene involvement, test drug molecules and study disease progression in animals, particularly mice and zebrafish, which are commonly used for cardiac research. Mouse models, due to their cardiac similarity to humans, have been immensely valuable in studying all aspects of AC, including gene involvement, specific human variants, signalling pathway interactions and disease progression. While whole-body or cardiac-specific KO mouse models have been useful, they often mimic conditions resembling non-sense mutations and truncating protein formation. However, homozygous and whole-body applications of this approach can lead to severe phenotypes and fetal mortality, and heterozygous models may not consistently exhibit a phenotype. The use of Tg-CS models, which overexpress human mutated proteins in cardiac tissue, has been employed to more faithfully reproduce phenotypes related to human mutations. However, this approach does not always mirror the effects observed in human conditions, especially due to the persistence of the functional ortholog in the transgenic animal. Studying human missense mutations in KI animal models, where the mutation is inserted into the endogenous gene, proves to be more effective, functioning through its own promoter. Notably, despite similarities, the human heart differs from the mouse heart, and characteristic features like the adipogenic substitution found in human ventricles are rarely observed in mouse models [116,129,231]. Conversely, the potential of the zebrafish model is evident, considering its economic, spatial, ethical, and genetic advantages, even though the development of zebrafish models for AC study was initially delayed, resulting in fewer stable mutant lines. Despite the challenge of duplicate gene copies, the zebrafish’s heart structure and protein organization allow the reproduction of a clear AC phenotype in generated models. While AC research in zebrafish is still in its early stages, with most models being transient KDs, the few stable models so far established have validated the reproducibility of the disease at both molecular and morphological levels. Additionally, drug testing is simpler, faster and more extensive due to the rapid development and the abundance of embryos obtained from each mating. These promising results suggest that zebrafish, in conjunction with mouse and in vitro models, could contribute significantly to a better understanding of AC, ultimately aiming to find a cure or alleviate severe phenotypic conditions observed in human patients.

## Figures and Tables

**Table 1:** Overview of non-desmosomal models with limited (yellow), disputed (orange), or not curated (green) evidence of association with AC.

GENE	MOUSE MODEL	ZEBRAFISH MODEL
LIMITED		
Sodium voltage-gated channel alpha subunit 5 (*SCN5A*) [189]	*CS-Scn5A*-KO mouse models showed severe biventricular cardiomyopathy and conduction defects in heterozygosity, whereas homozygotes can also present fetal lethality [190,191]. *Scn5A*-KI mouse models presenting human p.(Asp1275Asn) or p.Asp222Gln mutations showed a progressive cardiac dysfunction but not a clear AC morphological phenotype [192,193].	A CS-Tg zebrafish expressing the human Asp1275Asn mutation displayed bradycardia, conduction-system abnormalities and premature death, highlighting zebrafish as a valuable model for understanding the gene’s role in the disease [194]. Similarly, KD of *scn5a* in zebrafish delayed early chamber development and disrupted the ventricle’s patterned growth, suggesting that cardiac sodium channels influence heart development through a non-electrophysiological mechanism [195].
N-cadherin (*CDH2*) [196,197]	Ventricular arrhythmia and aberrant conduction in mice with CS-*Cdh2* deletion were observed, resulting in early death after two months. However, more studies are needed to understand CDH2’s role in the pathogenesis of the disease [198].	According to the ZFIN database, insertional, ENU- and CRISPR-induced mutants are available but not characterized as putative AC models.
Titin (*TTN*) [199]	Only after hemodynamic stress, the *Ttn* KI mouse model, mimicking the human p.(Met14544*) mutation, developed left ventricular dilatation, impaired fractional shortening and diffused myocardial fibrosis [200].	Homozygous N-terminal and C-terminal *titin* truncations led to severe cardiac contractility defects and premature death in zebrafish. C-terminal truncations caused severe skeletal muscle myopathies, while heterozygous truncations resulted in spontaneous DCM with decreased baseline ventricular systolic function, mirroring human conditions [201,202,203,204].
Lamin A/C (*LMNA*) [205]	Homozygous *Lmna*-KO mice died in a few weeks due to delayed postnatal development and the onset of DCM [206]. Heterozygous KO mutant mice, instead, survived, showing conduction defects, arrhythmia events and cardiac contractility problems [207]. KI mouse models expressing human mutations p(Arg225Ter) and p.(Arg541Cis) confirmed the conduction alterations and, in general, the phenotype observed in KO ones [208,209].	According to the ZFIN database, morphants, insertional, ENU- and CRISPR-induced mutants are available but not characterized as putative AC models.
Transforming growth factor β-3 (*TGFB3*) [210]	*Tgfβ3*-KO mice failed to display cardiac defects or AC phenotypes; therefore, this protein is currently more recognized for its indirect role in the disease, being found to be differently expressed in several other AC models [39,42,52,53,155].	According to the ZFIN database, morphants, ENU- and CRISPR-induced mutants are available but not characterized as putative AC models.
DISPUTED		
Cardiac Ryanodine receptor *2* (*RYR2*) [211]	RYR2 primarily impacts cardiac electrical activity rather than tissue remodelling. The KI mouse model with the human disease-associated *RYR2* mutation p.Arg176Gln exhibited calcium-dependent ventricular arrhythmias without histological changes [212], like other KI models [213,214] and a CS-KO one [215]. Whole body KO homozygosity provoked embryonic lethality [216].	According to the ZFIN database, ENU- and CRISPR-induced mutants are available but so far not characterized.
NOT CURATED		
Filamin C (*FLNC*) [217]	CS-*Flnc* deficient mice exhibited cardiac fibrosis, dysfunction, the elevated expression of cardiac stress markers and early mortality. These findings connected FLNC with the regulation of the cardiomyocyte structure [218,219,220].	Zebrafish *flnc* models (morphants and Tg) display cardiac alterations both functionally and structurally, with reduced survival. The observed effects included pericardial edema, dysmorphic or dilated cardiac chambers with protein aggregation, abnormal heart tube looping, reduced blood circulation and overall weaker contractility. TEM analysis revealed irregular or seemingly absent Z-discs [221,222].
Integrin-linked kinase (*ILK*) [223]	In mouse cardiomyocytes, *Ilk* deletion produced a lethal AC phenotype with relevant ion channel and structural remodelling, connecting this protein to the disease [224].	CS expression of human wild-type and mutated variants (H77Y and P70L) of *ILK* resulted in cardiac malfunction, decreased fractional shorting and premature mortality by the time the fish were two-to-three weeks old [223]. Likewise, a spontaneous homozygous *ilk* mutation (p.Leu308Pro) in zebrafish resulted in cardiac edema, reduced heart function, and early mortality [225,226].
Galectin-3 (*LGALS-3*/*GAL-3*) [227]	*Gal-3* emerged as one of the differentially expressed genes (DEGs) in the myocardium of Tg mice with the early AC phenotype. Dysregulation in the myocardium was found in Tg mice overexpressing the *DSG2* p.Asn271Ser mutation. This finding was further validated in three AC patients who experienced SCD without structural remodelling [227].	A pharmacological KD zebrafish *lgals3a* line exhibited developmental defects, decreased macrophages, apoptotic cardiomyocytes, and the dysregulation of the Wnt/β-catenin pathway, suggesting a potential role of *lgals3a* in the etiology of AC and other cardiac diseases [227,228].
Patatin Like Phospholipase Domain Containing 2 (*PNPLA2*) [229]	A KI mouse model carrying the human *Pnpla2* mutation presented with arrhythmias and significant cardiac dysfunction. Moreover, those mice suffered SCD with extensive lipogenesis in cardiomyocytes and cardiac fibrosis in the myocardium [229].	According to the ZFIN database, morphants and CRISPR-induced mutants are available but not characterized as putative AC models.
SH3 domain-containing 2b (*SORBS2*) [230]	KO mice with a complete SORBS2 depletion exhibited a phenotype resembling that of AC patients, featuring right ventricular dilatation, dysfunction, spontaneous ventricular tachycardia, and sudden cardiac death. The absence of SORBS2 caused significant cardiac electrical remodelling, affecting action potentials, impulse conduction, and inducing life-threatening arrhythmias [230].	According to the ZFIN database, morphants and mutants are available but not characterized as putative AC models.
